# Genetic Polymorphisms and the Risk of Accelerated Renal Function Decline in Women

**DOI:** 10.1371/journal.pone.0004787

**Published:** 2009-03-10

**Authors:** Cynthia Cooper Worobey, Naomi D. L. Fisher, David Cox, John P. Forman, Gary C. Curhan

**Affiliations:** 1 Renal Unit, Department of Medicine, Massachusetts General Hospital, Boston, Massachusetts, United States of America; 2 Endocrine-Hypertension Division, Department of Medicine, Brigham and Women's Hospital, Harvard Medical School, Boston, Massachusetts, United States of America; 3 Channing Laboratory, Department of Medicine, Brigham and Women's Hospital, Harvard Medical School, Boston, Massachusetts, United States of America; 4 Renal Division, Department of Medicine, Brigham and Women's Hospital, Harvard Medical School, Boston, Massachusetts, United States of America; 5 Department of Epidemiology, Harvard School of Public Health, Boston, Massachusetts, United States of America; Innsbruck Medical University, Austria

## Abstract

**Background:**

Reduced glomerular filtration rate is an important predictor of cardiovascular disease and death. Genetic polymorphisms, particularly in genes involved in the renin-angiotensin system (RAS), may influence the rate of renal function decline.

**Methodology/Principal Findings:**

We examined the relation between specific single nucleotide polymorphisms (SNPs), including those in the RAS, apolipoprotein E and alpha-adducin, and renal function decline assessed by estimated glomerular filtration rate (eGFR) over an 11-year period in 2578 Caucasian participants of the Nurses' Health Study. Logistic regression was used to examine the associations between genotype and risk of eGFR decline of ≥25%.

**Results:**

After 11 years between creatinine measurements, the eGFR declined by ≥25% in 423 of 2578 (16%) women. The angiotensinogen (AGT) A-20C polymorphism was associated with a higher risk of renal function decline when two risk alleles were present than if one or no alleles were present (CC vs AA and AC) OR 1.83 (95% CI 1.02–3.26; p = 0.04). The angiotensin II type 1 receptor (AT_1_R) A1166C polymorphism was marginally associated with a higher risk of renal function decline when two risk alleles were present (CC vs AA, OR = 1.41; 95% CI 0.98–2.01; p = 0.06). The alpha-adducin G460W polymorphism was associated with a lower risk of renal function decline when any number of risk alleles were present (WG vs GG, OR = 0.78, 95% CI 0.61–0.99, p = 0.04; WW vs GG, OR = 0.46; 95% CI 0.20–1.07, p = 0.07). Linear regression analysis with change in eGFR as the outcome showed a larger decline of 3.5 (95% CI 0.5 to 6.4, p = 0.02) ml/min/1.73 m^2^ in AGT A-20C CC homozygotes. No other polymorphisms were significantly associated with renal function decline or absolute change in eGFR over the study period.

**Conclusions:**

Genetic variants in the angiotensinogen, angiotensin II type 1 receptor and alpha-adducin genes may contribute to loss of renal function in the general female Caucasian population.

## Introduction

Reduced glomerular filtration rate is an important predictor of cardiovascular disease and death, in high-risk groups as well as in the general population [1], [2]. Even in the absence of identifiable risk factors for renal function decline, such as hypertension or diabetes, kidney dysfunction may develop slowly over decades. Genetic polymorphisms may confer increased risk of renal decline either through direct effects or by increasing susceptibility to environmental factors.

Blockade of the renin-angiotensin-system (RAS) [3]–[5] has been shown to slow renal function decline in individuals with renal disease, providing evidence that activation of the RAS may promote a more rapid loss of GFR. Because the activity of the RAS is intimately related to systemic blood pressure, dozens of studies have examined the relation between blood pressure or hypertension and polymorphisms in RAS-related genes, but the findings have been inconsistent [6], [7].

Polymorphisms in other genes, specifically alpha-adducin and apolipoprotein, may also promote renal function decline. Increased alpha-adducin activity influences sodium handling and glomerular hemodynamics in experimental animals and is associated with hypertension in humans [8], [9]. Conversely, compared to other apolipoprotein E types, the epsilon 4 variant lowers the risk of adverse renal outcomes [10]. Therefore, functional genetic variation in these genes may be involved in the loss of renal function over time.

Most prior investigations of these polymorphisms and renal function have focused on populations with established chronic kidney disease or heart disease. In contrast, their association with renal function decline in a general population without established renal disease has not been extensively studied. We examined these relations over an 11 year period in 2,578 Caucasian participants of the Nurses' Health Study.

## Results

The mean age of the women in this sub-cohort in 1989 was 56.3 years (median, 56; interquartile range, 51–62) and the mean BMI was 25.5 kg/m^2^ (median, 24.4; interquartile range 22.0–27.8). There were no significant differences in baseline age or BMI across any genotype (data not shown). The mean creatinine at baseline was 0.76 mg/dl and the mean eGFR at baseline was 86 ml/min/1.73 m^2^.

The genotype frequency for each polymorphism is shown in Table 1. Other than the ACE I/D polymorphism (p = 0.04), no other SNP deviated significantly from Hardy-Weinberg equilibrium.

**Table 1:** Genotype frequencies among 2578 Caucasian women from the Nurses' Health Study.

Genotype	Genotype frequency (%)						p-value*
**AGT M235T**	M/M		M/T		T/T		
	881 (34.2)		1184 ( 45.9)		443 (17.2)		0.19
**AGT A-20C**	A/A		A/C		C/C		
	1750 (67.9)		692 (26.8)		62 (2.4)		0.51
**ACE I/D**	I/I		I/D		D/D		
	515 (20.0)		1122 (43.5)		726 (28.2)		0.04
**AT_1_R A1166C**	A/A		A/C		C/C		
	1259 (48.8)		1017 (39.4)		233 (9.0)		0.18
**AS T-344C**	T/T		T/C		C/C		
	739 (28.7)		1182 (45.8)		531 (20.6)		0.15
**Adducin G460W**	G/G		G/W		W/W		
	1709 (66.3)		707 (27.4)		67 (22.6)		0.59
**APOE C334T**	C/C		C/T		T/T		
	54 (2.1)		594 (23.0)		1860 (72.1)		0.42
**APOE C472T**	C/C		C/T		T/T		
	2126 (82.5)		331 (12.8)		18 (0.7)		0.20
**APOE (Summ. Score)**	E2/E2 (−2)	E3/E2 (−1)	E4/E2 (0)	E3/E3 (0)	E3/E4 (+1)	E4/E4 (+2)	
	18 (0.7)	273 (10.6)	58 (2.2)	1537 (59.6)	528 (20.5)	46 (1.8)	

Totals do not equal 2578 because of variable numbers with missing genotypes.

* Chi-square test for deviation from Hardy-Weinberg equilibrium.

ACE, Angiotensin Converting Enzyme.

AGT, Angiotensinogen.

AT_1_R, Angiotensin II Receptor Type 1.

AS, Aldosterone Synthase.

APOE, apolipoprotein E.

During 11 years between creatinine measurements, the eGFR declined 25% or greater in 423 of 2578 (16%) women. Age-adjusted associations between polymorphisms and a ≥25% decline in eGFR are shown in Table 2. The angiotensinogen AGT A-20C polymorphism was associated with a higher risk of renal function decline when two risk alleles were present than if one or no alleles were present (CC vs AA and AC) OR 1.83 (95% CI 1.02–3.26; p = 0.04). The angiotensin II receptor type 1 (AT_1_R) A1166C polymorphism was marginally associated with a higher risk of renal function decline when two risk alleles were present (CC vs AA, OR = 1.41; 95% CI 0.98–2.01; p = 0.06). The alpha-adducin G460W polymorphism was associated with a lower risk of renal function decline when any number of risk alleles were present (WG vs GG, OR = 0. 78, 95% CI 0.61–0.99; p = 0.04; WW vs GG, OR = 0.46; 95% CI 0.20–1.07; p = 0.07); the OR for the dominant model was 0.75 (95% CI 0.59–0.95; p = 0.02). Linear regression analysis with change in eGFR as the outcome showed a larger decline of 3.5 (95% CI 0.5 to 6.4; p = 0.02) ml/min/1.73 m^2^ in AGT A-20C CC homozygotes. No other polymorphisms were significantly associated with renal function decline or absolute change in eGFR over the study period.

**Table 2:** Association between Gene Polymorphisms and Risk of ≥25% Decline in GFR Over 11 Years, Adjusted for Age.

	Dose Response Model (OR, 95%CI)	Dominant Model (OR, 95%CI)	Recessive Model (OR, 95%CI)
**AGT M235T**	M/T: 1.02 (0.81–1.30)	1.00 (0.80–1.24)	0.91 (0.69–1.21)
	T/T: 0.92 (0.68–1.27)		
**AGT A-20C**	A/C: 0.99 (0.78–1.26)	1.05 (0.84–1.32)	1.83 (1.02–3.26)
	C/C: 1.82 (1.02–3.27)		
**ACE I/D**	I/D: 0.87 (0.66–1.15)	0.90 (0.69–1.16)	1.03 (0.81–1.30)
	D/D: 0.94 (0.69–1.26)		
**AT_1_R A1166C**	A/C: 1.17 (0.94–1.47)	1.22 (0.98–1.50)	1.31 (0.93–1.84)
	C/C: 1.41 (0.98–2.01)		
**AS T-344C**	T/C: 0.86 (0.68–1.11)	0.87 (0.70–1.10)	0.97 (0.75–1.26)
	C/C: 0.89 (0.66–1.20)		
**Adducin G460W**	G/W: 0.78 (0.61–0.99)	0.75 (0.59–0.95)	0.49 (0.21–1.14)
	W/W: 0.46 (0.20–1.07)		
**APOE**	Relative Risk per Unit Increase in summary score: 1.08 (0.91–1.28)		

AGT, Angiotensinogen; reference = M/M (dose response and dominant models) or M/M+M/T (recessive model).

AGT, Angiotensinogen; reference = A/A (dose response and dominant models) or A/A+A/C (recessive model).

ACE, Angiotensin Converting Enzyme; reference = I/I (dose response and dominant models) or I/I+I/D (recessive model).

AT_1_R, Angiotensin II Type 1 Receptor; reference = A/A (dose response and dominant models) or A/A+A/C (recessive model).

AS, Aldosterone Synthase; reference = T/T (dose response and dominant models) or T/T+T/C (recessive model).

Adducin G460W; reference = G/G (dose response and dominant models) or G/G+G/W (recessive model).

APOE, Apolipoprotein A; reference = APOE Summary Score 0; Summary Score system respectively assigns +1, 0, or −1 per E2, E3, or E4 allele of an individual with genotypes (and scores) of: E2/E2 (+2), E2/E3 (+1), E2/E4 (0), E3/E3 (0), E3/E4 (−1), E4/E4 (−2).

We explored potential interactions with BMI and history of hypertension. Only the ORs for dominant models for alpha-adducin G460W varied by BMI and HTN. The OR for eGFR decline ≥25% was 0.99 (0.72–1.35) for BMI<25 kg/m^2^ and 0.52 (0.36–0.77) for BMI≥25 (p, interaction = 0.06). The OR for eGFR decline ≥25% was 0.65 (0.49–0.88) for no history of hypertension and 0.99 (0.65–1.49) for a history of hypertension (p, interaction = 0.03).

## Discussion

We found several polymorphisms associated with altered risk of renal function decline over an 11-year period. Homozygosity for the angiotensinogen AGT A-20C polymorphism was associated with an 83% increased odds of a ≥25% decline in eGFR over 11 years. Homozygosity for the AT_1_R A1166C polymorphism was marginally associated with a 41% increased odds of a ≥25% decline in eGFR over 11 years. The alpha-adducin G460W polymorphism was associated with a 25% decreased odds of a ≥25% decline in eGFR over 11 years.

Genetic susceptibility may play an important role in rate of renal function decline. Clustering of renal disease occurs within families, with up to 30% of patients with ESRD having an affected sibling [11]–[15]. Like many complex disorders, it is unlikely that a single genetic polymorphism will explain all susceptibility to accelerated renal function decline. However, certain genetic alterations that affect the expression or function of a key protein product, so called functional polymorphisms, may influence pathological processes either promoted or prevented by that key protein [16]. Randomized controlled trials with agents that interrupt the renin-angiotensin-system (RAS) [3]–[5] have demonstrated a slowing of renal function decline in individuals with renal disease, providing strong evidence that activation of the RAS may promote a more rapid loss of GFR in patients with renal disease. While speculative, it is intriguing to consider the possibility that more directed pharmacologic therapy might derive from findings like these; for example, blockade of the AT_1_R with angiotensin receptor blockade might be especially beneficial in these patients [17].

Polymorphisms in the angiotensinogen gene (M235T and A-20C) have been associated with hypertension [18], [19], faster progression to ESRD [20], susceptibility to nephropathy in patients with type I diabetes mellitus [21], and progression of renal dysfunction in adults [22] and children [23] with IgA nephropathy. There was no association found in two populations with type II diabetes [24], [25]. We found an association between the A-20C polymorphism and increased risk of renal function decline and no association with the other angiotensinogen polymorphism in our population.

Angiotensin II type 1 receptor (AT_1_R) polymorphisms may influence intrarenal angiotensin II activity. Healthy Caucasian carriers of the A1166C polymorphism showed 7% lower basal GFR and 17% lower basal renal plasma flow, as well as enhanced increases in GFR following treatment with the AT_1_R blocker losartan [17]. Rate of progression to ESRD in patients with nephropathies of various etiologies was more rapid in individuals homozygous for the AT_1_R A1166C polymorphism [26], [27]. Our study found a similar association between homozygosity for the AT_1_R A1166C polymorphism and an accelerated rate of renal function decline.

The deletion polymorphism in intron 16 of the ACE gene has been correlated with increased plasma ACE activity [28]. An increased risk of progression of renal disease associated with the ACE-D allele has been reported in some populations with renal disease [20], [29] but not in others [30], [31]. A genetic predisposition to diabetic nephropathy based on the DD genotype has been reported in several studies [32]–[34], but remains controversial [35]. We found no association between the insertion/deletion polymorphism of the ACE gene and accelerated renal function decline.

Aldosterone, independent of angiotensin II, has been associated with renal dysfunction and glomerulosclerosis in remnant kidney rat models and glomerular hyperfiltration in humans with primary aldosteronism [36], [37]. Past studies of aldosterone synthase gene polymorphisms and hypertension have been mixed [38]–[41] and no prior study has found an association between the aldosterone synthase polymorphism and progression of renal disease [20]. We similarly found no association with the T-344C aldosterone synthase polymorphism and accelerated renal function decline.

Adducin is a membrane cytoskeleton-associated protein consisting of an alpha subunit and either a beta-or gamma-subunit that promotes the assembly of the spectrin-actin network. In rats and humans, mutations of the alpha-adducin subunit lead to the stimulation of Na(+), K(+)-ATP-ase activity in renal tubular cells, increased renal Na(+) reabsorption and, subsequently, low-renin hypertension [8], [9]. A familial aggregation study demonstrated that low renin hypertension was associated with the alpha-adducin G460W polymorphism [42]. In a study of 260 ESRD patients matched to controls, the time from diagnosis with renal disease to onset of ESRD was shorter for patients homozygous for tryptophan (Trp) at the glycine to tryptophan (G460W) polymorphism versus those homozygous for glycine (Gly) [43]. The alpha-adducin Trp polymorphism has been associated with lower GFR in essential hypertensive individuals when on a low sodium diet but not when on a normal-high sodium diet [44]. It was postulated that the Trp polymorphism may be associated with increased GFR which balances the increased sodium reabsorption [45]. This hyperfiltration might lead to steeper decline in renal function over time, especially in the presence of renal disease. In contrast, we found an apparent protective effect of the alpha-adducin Trp polymorphism on renal function decline. Perhaps this polymorphism, which confers a greater sodium resorptive capacity, may protect against volume depletion and subsequent stimulation of the RAS system.

Epsilon 4 apolipoprotein is defined by the presence of both the 344C and 472C polymorphisms in the APOE gene. This variant has been associated with a reduced risk of adverse renal outcomes when compared with Epsilon 3 (334T, 472C) and Epsilon 2 (334T, 472T) apolipoprotein, including a 40% reduction in risk for the development of chronic kidney disease (RR = 0.60, 95% CI 0.43–0.84) in the Atherosclerosis Risk in Communities (ARIC) study [10]. Also, the time lag from identifiable onset of diabetes to initiation of permanent hemodialysis was two-fold higher in apoE4 carriers than the rate in non-apoE4 carriers among non-insulin dependent diabetics [46]. The frequencies of apolipoprotein E variants in our population were similar to the ARIC population. However, the ARIC study found the strongest associations between apoE type and their endpoint, progression of chronic kidney disease, among the African American subgroup, with a non-significant result for the Caucasians. Our analysis used the summary score schema from the ARIC study, but we found that risk of renal function decline did not significantly increase with increasing summary score. The null findings in our primarily Caucasian population more closely reflect the results of the ARIC Caucasian subgroup. Also, the endpoint analyzed for ARIC study was determined by ICD coding of hospitalizations, rather than a measured creatinine value, for over half (55.7%) of their subjects with this outcome. The outcome in our study was strictly based on eGFR change over time and may in part account for our different results.

Because our participants were all female and Caucasian, the genotype frequencies and results may not be generalizable to other populations. Our population is generally healthy and has a low rate of both baseline chronic kidney disease and diabetes, two groups in which much of the prior genetic studies of renal outcomes had been performed. The outcome was based on only two measurements of serum creatinine separated by 11 years. Although there was likely misclassification of GFR due to limitations of the MDRD equation in individuals with near normal renal function, our study was large and the duration of follow-up was long; therefore, it is unlikely that we missed large associations. A third creatinine measurement would reduce variability in the estimate of change in renal function over time. Larger studies will be needed to examine gene-gene and gene-environment interactions.

In contrast to many genetic studies (such as genome wide association studies), we selected these genes with specific *a priori* hypotheses and thus believe it is justified to use the traditional level of statistical significance (p<0.05). We acknowledge, however, that in the absence of highly statistically significant results (on the order of p<10^−3^), these associations should be carefully interpreted, and replication in separate studies is necessary. The results would no longer be considered statistically significant if the p-values were adjusted for multiple comparisons.

### Conclusion

Genetic polymorphisms in the angiotensinogen, angiotensin type 1 receptor and alpha-adducin genes were associated with the rate of renal function decline in our population. Other genetic variants, including the ACE insertion/deletion, were not associated with renal function decline. Genetic variants may contribute to loss of renal function in the general female Caucasian population.

## Methods

### Study Population

The Nurses' Health Study (NHS) began in 1976, when 121,700 female nurses 30 to 55 years of age completed a detailed questionnaire regarding health-related information. Since then, subsequent questionnaires updated information on lifestyle factors and new medical diagnoses every 2 years. The participants in the current study were part of a substudy designed to assess the association between analgesic use and change in renal function [47]. Briefly, we limited our study sample to the 32,826 participants who provided a blood sample in 1989. Women were excluded from this initial blood collection if they had a history of either cancer (except non-melanoma skin cancer) or cardiovascular disease (myocardial infarction, angina, stroke, or transient ischemic attack). The characteristics of the women who provided blood samples were similar to those of the total cohort in terms of prevalent hypertension, age, weight, diabetes mellitus, and hyperlipidemia, but those who provided blood samples were less likely to be active smokers.

The population was then further limited to 3,123 women who answered a supplementary questionnaire about analgesic use and who provided a second blood sample in 2000, and to 2691 women who had serum creatinine measured on both blood samples. The population was further restricted to Caucasians in an effort to limit population admixture, leaving a final study population of 2578 women. The institutional review board at Brigham Women's Hospital approved this study. No written informed consent was required for this study by the IRB.

### Blood Collection

Each blood collection kit contained all the necessary instructions and supplies to have blood drawn and mailed back to our laboratory with an ice pack. The samples were returned to our laboratory via overnight courier; over 95% of the samples arrived within 24 hours of being drawn. Upon arrival in our laboratory, the chilled blood samples were centrifuged in a refrigerated unit and blood components aliquotted in cryotubes that are stored in the vapor phase of liquid nitrogen freezers; the highest temperature is −130°C. After extraction, the DNA obtained from the buffy coats is well preserved. Neither the transport nor the delay in processing appreciably decreased the amount of DNA recovered [48].

### Genotyping

Polymorphisms were selected because of their relation to the RAS or proposed association with renal function decline. These included two angiotensinogen polymorphisms, AGT M235T (rs699) and AGT A-20C (rs5050), the angiotensin converting enzyme insertion/deletion polymorphism (ACE I/D, no rs number), the angiotensin II type 1 receptor A1166C polymorphism (AT_1_R A1166C, rs5186), and the aldosterone synthase T-344C polymorphism (AS T-344C, rs1799998). All women in the study were also genotyped for the alpha adducin G460W polymorphism (rs4961) and the APOE C334T (rs429358) and C462T (rs7412) polymorphisms, which determine apolipoprotein E types 2, 3, or 4. Genotyping was performed at the Harvard/Partners Genotyping Facility using Taqman or Sequenom. Failure to genotype occurred to varying degrees, but none more than 8.3 %, which was the failure rate for ACE I/D. We tested for deviations from Hardy-Weinberg equilibrium for each SNP using the Chi-square test and there were no substantial deviations (P≥0.04; Table 1).

### Assessment of Renal Function

In 2001, baseline and follow-up blood samples from 1769 participants were thawed and sent for measurement of creatinine. Both samples from each woman were run in the same laboratory batch. Creatinine was measured by a Hitachi autoanalyzer using a modified kinetic Jaffe reaction. The overall coefficient of variation of the masked quality control samples was 10%. In 2005, additional blood samples were thawed and sent for measurement of creatinine using the above protocol to achieve the study population of 2578 Caucasian women. The overall coefficient of variation for creatinine of masked quality control samples for this group was also 10%.

Glomerular filtration rate was estimated using the Modification of Diet in Renal Disease (MDRD) formula [49]. The simplified MDRD formula has been found to vary most from measured GFR in individuals with normal or near normal renal function, i.e. ≥60 ml/min/1.73 m^2^. However, given the impracticalities of directly measuring GFR in large cohort studies, the MDRD formula appears to provide the best available estimate and has been used successfully in several studies [50], [51]. The MDRD formula for Caucasians is creat^−1.154^*age^−0.203^*0.742.

### Statistical analysis

Estimated GFR (eGFR) was calculated for each woman at the beginning and end of the 11 year time period. The primary outcome was a ≥25% decline in eGFR from baseline. Logistic regression was used to examine the associations between genotype and risk of eGFR decline of ≥25%. The secondary outcome was absolute change in eGFR from baseline. Linear regression was used to examine the associations between genotype and this change.

APOE variation was modeled with a summary score model, which had been used in a prior study of genotype and chronic kidney disease [10]. This genotypic scoring system respectively assigns +1, 0, or −1 per E2, E3, or E4 allele of an individual with genotypes (and scores) of: E2/E2 (+2), E2/E3 (+1), E2/E4 (0), E3/E3 (0), E3/E4 (−1), E4/E4 (−2). Summary scores increase power in modeling genetic exposures [52]. The reference group for the APOE analysis was summary score 0. For all other polymorphisms, the genotype that *a priori* was hypothesized to confer the lowest risk of renal function decline was used as the reference group. For example, if for an allele p/q the q allele was hypothesized to be associated with faster renal function decline, then the p allele was used as the referent group. Genotype – renal function associations were analyzed in three ways: per additional risk allele (3 categories including pp as the reference group, pq, and qq); recessive model (pp and pq combined as the reference group); and a dominant model (pp as the reference group, with pq and qq combined as the comparison group). Possession of a particular polymorphism was assumed to be an inherent attribute that would not be confounded by dietary or demographic factors. Given this assumption, the primary models were adjusted for age alone. However, genotypes that influence the risk of hypertension could also influence the rate of renal function decline, thus hypertension could be along the causal pathway between genotype and rate of renal function decline. We therefore performed additional analyses that adjusted for hypertension, as well as diabetes mellitus and smoking. No adjustment was made for multiple comparisons because each SNP was selected with an *a priori* hypothesis. Odds ratios (OR) and 95% confidence intervals (95% CIs) were calculated for all polymorphisms.

We also examined potential interactions with BMI (<25 kg/m^2^ and ≥25) and history of hypertension (yes/no) [53].
